# Comprehensive transcriptomic analysis of immune-related eRNAs associated with prognosis and immune microenvironment in melanoma

**DOI:** 10.3389/fsurg.2022.917061

**Published:** 2022-09-09

**Authors:** Yuling Gan, Yuan Yang, Yajiao Wu, Tingdong Li, Libing Liu, Fudong Liang, Jianghua Qi, Peng Liang, Dongsheng Pan

**Affiliations:** ^1^ The 1st Department of Bone and Soft Tissue Oncology, Gansu Provincial Cancer Hospital, Lanzhou, China; ^2^ Department of Gastroenterology, The First Hospital of Lanzhou University, Lanzhou, China; ^3^ Department of Ophthalmology, The First Hospital of Lanzhou University, Lanzhou, China

**Keywords:** eRNA, tumor immune, prognosis, melanoma, corticosterone

## Abstract

**Background:**

Recent evidence suggests that enhancer RNAs (eRNAs) play key roles in cancers. Identification of immune-related eRNAs (ireRNAs) in melanoma can provide novel insights into the mechanisms underlying its genesis and progression, along with potential therapeutic targets.

**Aim:**

To establish an ireRNA-related prognostic signature for melanoma and identify potential drug candidates.

**Methods:**

The ireRNAs associated with the overall survival (OS-ireRNAs) of melanoma patients were screened using data from The Cancer Genome Atlas (TCGA) *via* WGCNA and univariate Cox analysis. A prognostic signature based on these OS-ireRNAs was then constructed by performing the least absolute shrinkage and selection operator (LASSO) Cox regression analysis. The immune landscape associated with the prognostic model was evaluated by the ESTIMATE algorithm and CIBERSORT method. Finally, the potential drug candidates for melanoma were screened through the cMap database.

**Results:**

A total of 24 OS-ireRNAs were obtained, of which 7 ireRNAs were used to construct a prognostic signature. The ireRNAs-related signature performed well in predicting the overall survival (OS) of melanoma patients. The risk score of the established signature was further verified as an independent risk factor, and was associated with the unique tumor microenvironment in melanoma. We also identified several potential anti-cancer drugs for melanoma, of which corticosterone ranked first.

**Conclusions:**

The ireRNA-related signature is an effective prognostic predictor and provides reliable information to better understand the mechanism of ireRNAs in the progression of melanoma.

## Introduction

Melanoma is the most aggressive form of skin cancer, responsible for 90% of skin cancer-related death (1). While complete surgical resection is curative for early melanoma, it is largely ineffective against the metastatic cancer (2). In addition, targeted therapies have also not been able to improve survival outcomes of patients with metastatic melanoma (3). Accurate assessment of melanoma prognosis is crucial to guide clinical decision-making. To date, there are no highly sensitive and accurate prognostic biomarkers for melanoma. Given this, identification of novel biomarkers with prognostic and therapeutic significance is urgently needed.

Currently, melanoma staging is based on the American Joint Committee on Cancer (AJCC) melanoma TNM staging system, and is used by clinicians to assess prognosis and establish a treatment regimen (4). However, this system has its shortcomings and cannot meet the need for precision medicine. To improve the accuracy in assessing the melanoma prognosis, we need more objective methods. Recent evidence is accumulating on promising biomarkers, including enhancer RNAs (eRNAs) (5, 6).

Enhancers are principal gene regulatory elements that control transcription of linked genes (7). Studies increasingly show that enhancers also transcribe long noncoding RNAs (lncRNAs), known as the enhancer RNAs or eRNAs (8, 9), that were initially considered as transcriptional by-products. However, emerging evidence supports that most eRNAs are not transcriptional byproducts and play crucial roles in transcriptional activation and regulation of chromatin modeling (10). Previous studies have shown their direct involvement in tumorigenesis of cancers (11). Bal et al. found that mutations located in transcribed sequences encoding eRNAs impaired enhancer activity and ACTRT1 expression, which was instrumental in the initiation of basal cell carcinoma (12). Furthermore, the eRNA AP001056.1 was reported to be associated with overall survival (OS) in patients with head and neck carcinoma (13). Together these findings reveal that eRNAs play crucial roles in cancers and could serve as potential therapeutic targets. Identifying eRNAs from active enhancers enabled us to understand deeper complexity of the transcription program in cancers. However, eRNAs with functional significance in melanoma remain largely elusive.

Compared to non-cutaneous melanoma, cutaneous melanoma has the highest genomic mutational load, which translates to increased immunogenicity (14) and potentially greater responsiveness to immunotherapies. In fact, antagonists of immune checkpoint molecules such as cytotoxic T-lymphocyte-associated protein 4 (CTLA-4) and programmed cell death protein 1 (PD-1) have been used for the treatment of unresectable or metastatic melanoma (15). However, a significant subset of melanoma patients are either unresponsive to these drugs or eventually develop resistance (16). Therefore, it is essential to explore the molecular mechanisms associated with melanoma genesis and immune evasion in order to identify more effective targets for immunotherapies. A recent study showed that eRNAs are involved in the activation of immune responses (17). Additionally, most causal variants in autoimmune diseases located in immune cell enhancers that produced eRNAs under immune stimulation (18). Zhang et al. found that the expression levels of immune checkpoints in cancer cells correlate with eRNAs (19). Although these findings strongly indicate a functional interaction between eRNAs and tumor immune, it remains to be ascertained whether it influences the genesis and progression of melanomas.

In the present study, we identified immune-related eRNAs (ireRNAs) with prognostic significance using The Cancer Genome Atlas (TCGA) database. Subsequently, a prognostic signature based on these ireRNAs was established and validated in two separate subsets, which showed a good performance in predicting the OS of patients with melanoma. We also explored the underlying mechanisms by performing functional enrichment analyses and assessing the characteristics of tumor immune in the high- and low-risk groups. Finally, we identified 1,309 potential drug candidates for melanoma using the Connectivity Map (cMap) database, of which corticosterone was ranked first (Supplementary Table S1).

## Methods

### Identification of survival-related immune genes

**Figure 1:**
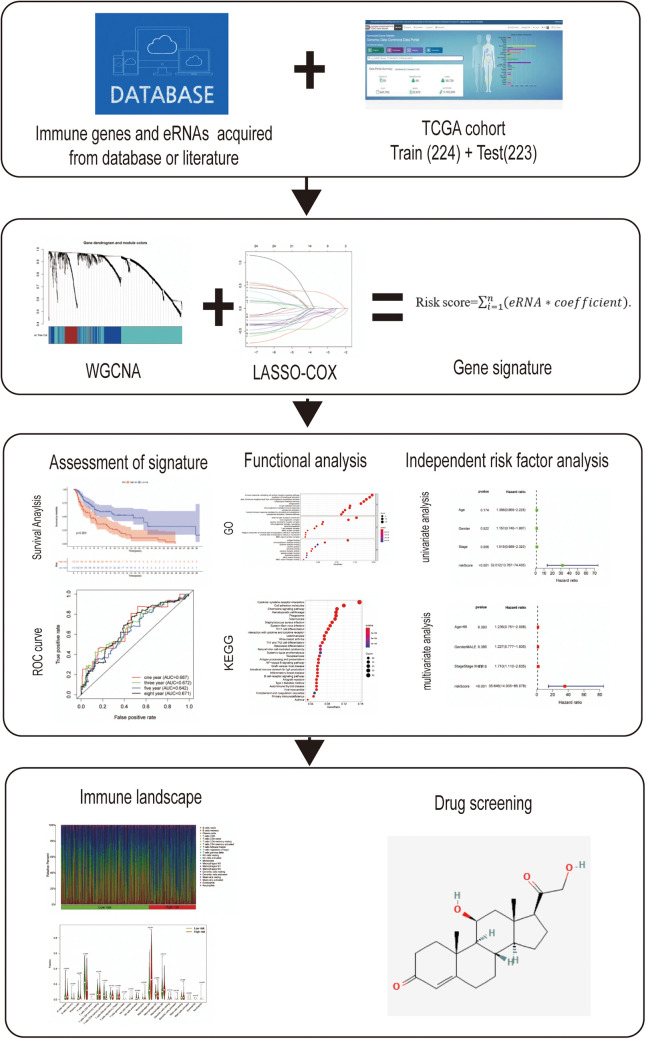
Flow diagram of the study.

The RNA-Seq transcriptome data and corresponding clinical information regarding cutaneous melanoma were downloaded from the TCGA data portal (https://tcga-data.nci.nih.gov/tcga/). After excluding samples from patients lacking complete survival information, a total of 447 samples were included in this analysis. These samples were randomly divided into a training data set (*n* = 224) and testing set (*n* = 223) as 1:1 rate using “caret” R package. Meanwhile, 1,811 immune-related genes were acquired from the ImmPort database (http://www.immport.org). Weighted gene co-expression network analysis (WGCNA) was performed to identify the prognosis-related modules in cutaneous melanoma using the “WGCNA” R package. These genes in the module were identified as survival-related immune genes and incorporated in subsequent analyses. The flow diagram of the study is shown in Figure 1.

### Construction of the prognostic signature based on survival-related ireRNAs

A total of 1,580 eRNAs were identified in cutaneous melanoma using the PreSTIGE algorithm as previously described (20), and the ireRNAs were further screened using correlation analysis with correlation coefficient >0.4 and *P*-value <0.05 as the thresholds (21). After performing univariate Cox regression analysis, those ireRNAs with *P*-value of <0.05 were identified as OS-associated ireRNAs (OS-ireRNAs) and included subsequent analysis. The ireRNAs with the highest correlation to OS were then screened by least absolute shrinkage and selection operator (LASSO) analysis according to the optimal penalty parameter (*λ*) value determined by 10-round cross-validation. These filtered OS-ireRNAs were incorporated into the multivariate Cox regression model to establish the prognostic signature. The following formula to calculate the risk score of each patient is: $\mathrm{Risk}\;\mathrm{score}=\sum_{i=1}^{n}(\mathrm{expression}\;\mathrm{level}\;\mathrm{of}\;\mathrm{eRNA}\;*$ LASSO regression coefficient). The median risk score among these patients in train cohort was used as the cut-off value. According to the median risk score, patients with cutaneous melanoma were divided into the high- and low-risk groups. To assess the prognostic value of the ireRNA-related signature, Kaplan–Meier survival curves were constructed to compare OS between the high- and low-risk groups using the “survival” and “survminer” R packages. And the log-rank test was used to assess whether they are statistically different. the prognostic performance of the model was determined by measuring the area under the receiver operating characteristic curve (AUC-ROC) with the “timeROC” R package. Univariate and multivariate Cox regression analyses were used to evaluate the relationship between OS and risk score and clinical characteristics. *P*-values <0.05 were regarded as statistically significant.

### Analyses of the immune landscape

The “estimate” R package was used to calculate the ratio of immune-stromal components in the tumor microenvironment for each melanoma sample, and to compared the differences in ESTIMATE score, stromal score, and immune score between the high- and low-risk groups. The relative proportions of 22 infiltrating immune cell populations in the two risk groups were evaluated according to the gene expression profile using the CIBERSORT computational method.

### Functional enrichment analyses

The differentially expressed genes (DEGs) between the high-risk and low-risk groups were screened according to absolute fold change (log2) >1.5 and FDR <0.05 as the thresholds. The DEGs were functionally annotated by Gene Ontology (GO) and Kyoto Encyclopedia of Genes and Genomes (KEGG) analyses using the “clusterProfiler” R package.

### Exploration of potential drugs for melanoma

To select potential drugs for cutaneous melanoma, the filtered list of DEGs between the high-risk and low-risk groups were utilized to query the cMap database (http://cmap-online.org/).

## Results

### Construction and validation of an ireRNA-related signature for melanoma

**Figure 2:**
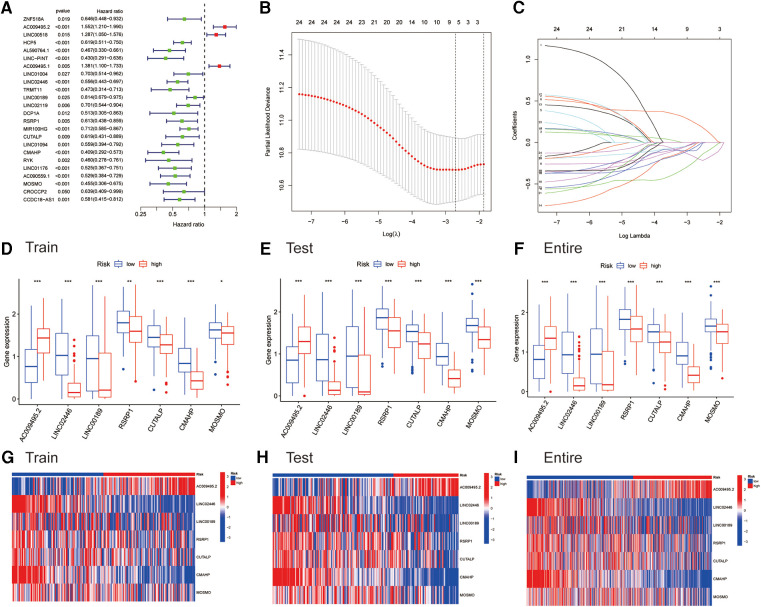
Identifying immune-related eRNAs (ireRNAs) associated with melanoma prognosis for construction of a signature. (**A**) Forest plots for hazard ratios of overall survival-related ireRNAs. (**B**) LASSO Cox analysis revealed 7 ireRNAs strongly correlated with the prognosis of melanoma. (**C**) The model’s penalty parameter (*λ*) was determined by 10-round cross-validation. (**D–F**) Differentially expressed analyses for ireRNAs between high- and low-risk groups in the training, testing, and entire cohorts, respectively. (**G–I**) Heatmap for ireRNAs involved in the signatures of the training, testing, and entire cohorts, respectively.

As previously mentioned, the entire cohort was divided into a training cohort (*n* = 224) and a testing cohort (*n* = 223). The detailed clinical characteristics of training cohort, testing cohort and entire cohort are summarized in Table 1. The training cohort was utilized to construct the model. A total of 353 immune-related genes involved in the MEblue module were characterized as survival-related immune genes and rolled into subsequent analyses (Supplementary Figure S1). We identified 33 ireRNAs, of which 24 were significantly correlated to the OS (Figure 2A). Subsequently, the LASSO regression analysis was performed to select the key OS-ireRNAs as candidates (Figures 2B,C). Finally, we built the prognostic signature with 7 OS-ireRNAs. The risk score of every patient was computed based on the following formula: risk score = AC009495.2 × 0.2300 + LINC02446 × (−0.2078) + LINC00189 × (−0.0083) + RSRP1 × (−0.0088) + CUTALP × (−0.0454) + CMAHP × (−0.1873) + MOSMO × (−0.0942). The median risk score was calculated as 0.817. According to the median risk score, patients with cutaneous melanoma in three cohorts (training cohort, testing cohort and entire cohort) were divided into the high- and low-risk groups, respectively. As shown in Figures 2D–F, there were significant differences in the expression of 7 OS-ireRNAs between the high-risk and low-risk groups in each cohort. Moreover, the heatmaps of these OS-ireRNAs expression profiles are shown in Figures 2G–I.

**Table 1:** Clinical characteristics of the melanoma patients used in the present study.

Clinical characteristics	Entire cohort	Train cohort	Test cohort
No. of patients	447	224	223
Age			
≤65	294 (65.77%)	146 (65.18%)	148 (66.37%)
>65	153 (34.23%)	78 (34.82%)	75 (33.63%)
Gender			
Female	168 (37.58%)	92 (41.07%)	76 (34.08%)
Male	279 (62.42%)	132 (58.93%)	147 (65.92%)
T			
T0–2	140 (31.32%)	61 (27.23%)	79 (35.43%)
T3–4	233 (52.13%)	126 (56.25%)	107 (47.98%)
Tis	7 (1.57%)	5 (2.23%)	2 (0.9%)
Tx	67 (14.99%)	32 (14.29%)	35 (15.7%)
N			
N0	222 (49.66%)	112 (50%)	110 (49.33%)
N1–3	176 (39.37%)	87 (38.84%)	89 (39.91%)
NX	49 (10.96%)	25 (11.16%)	24 (10.76%)
M			
M0	402 (89.93%)	205 (91.52%)	197 (88.34%)
M1	21 (4.7%)	11 (4.91%)	10 (4.48%)
Mx	24 (5.37%)	8 (3.57%)	16 (7.17%)

**Figure 3:**
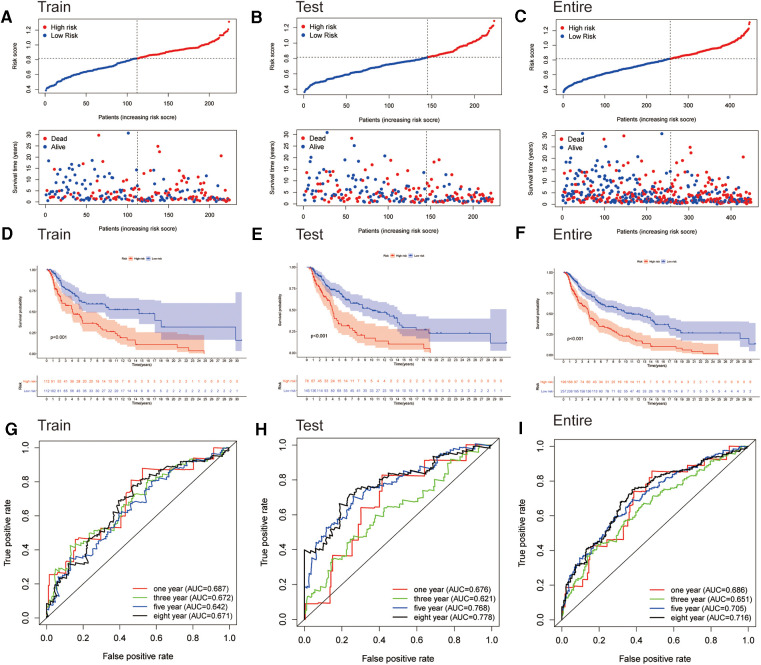
Assessment of the prognostic signature for melanoma. (**A–C**) Distributions of risk scores and OS status in the training, testing, and entire cohorts, respectively. (**D–F**) Kaplan-Meier survival analyses of the signature for the training, testing, and entire cohorts, respectively. (**G–I**) ROC curve of the prognostic signature in the training, testing, and entire cohorts, respectively.

We generated the figures to show the risk score and survival status of each cutaneous melanoma sample (Figures 3A–C). The results indicated that the outcomes of patients in the high-risk group were worse compared to those in the low-risk group. Furthermore, the high-risk group had significantly shorter OS compared to the low-risk group in the training cohort (Figure 3D). Consistent results were observed in the testing cohort as well as the entire cohort (Figures 3E,F). The AUC-ROC values of the prognostic signature for 1-, 3-, 5- and 8-year OS in the training cohort were 0.687, 0.672, 0.642 and 0.671 respectively (Figure 3G). The corresponding values were 0.676, 0.621, 0.768 and 0.778 in the test cohort, and 0.686, 0.651, 0.705 and 0.716 in the entire cohort (Figure 3H,I). Therefore, the above results showed that the established signature expressed a good performance in monitoring survival and was robust when validated in another cohort.

### The ireRNA-related signature independently predicts OS

**Figure 4:**
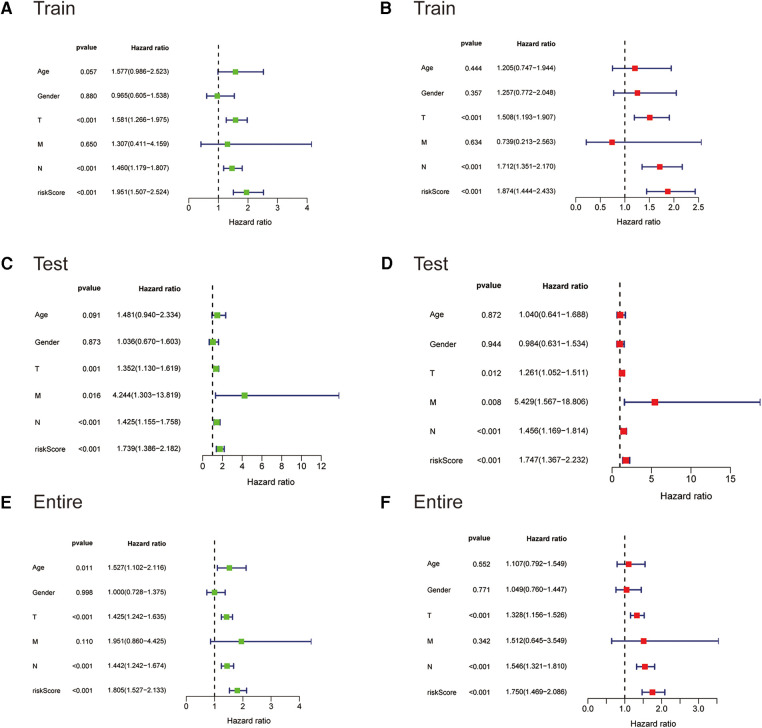
The Cox regression analyses for evaluating the independent prognostic value of the risk score. (**A,C,E**) Univariate Cox regression analyses of the association between survival and clinicopathological parameters and risk score for the training, testing, and entire cohorts, respectively. (**B,D,F**) Multivariate Cox regression analyses of the association between survival and clinicopathological parameters and risk score for the training, testing, and entire cohorts, respectively.

We next subjected the ireRNA signature risk score and other clinicopathological parameters, including age, gender and TNM stage, to Cox regression analyses to further identify the independent risk factors of cutaneous melanoma. According to the univariate analysis, the risk score was significantly correlated with the OS in the training cohort (Figure 4A). The multivariate analysis further identified the risk score, T stage and N stage as independent risk factors for cutaneous melanoma (Figure 4B). These findings were verified in the test cohort and the entire cohort (Figures 4C–F).

### Immune landscape of the ireRNA-related signature

**Figure 5:**
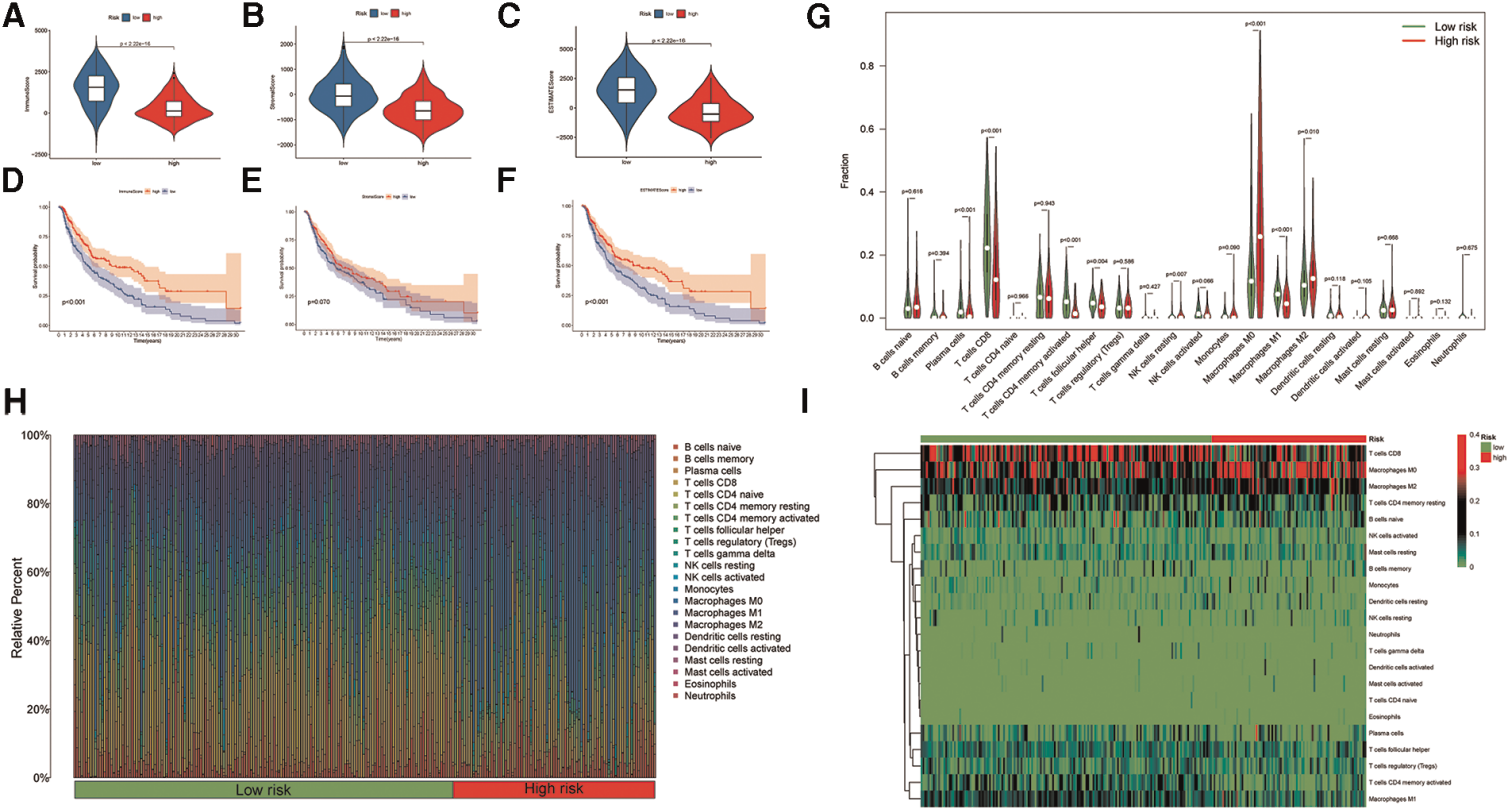
Association between the signature and immune landscape in melanoma. (**A–C**) The difference in immune, stromal, and ESTIMATE scores between the high- and low-risk groups. (**D–F**) Kaplan-Meier survival analyses of the risk scores based on the immune, stromal, and ESTIMATE scores, respectively. (**G**) Comparison of the infiltrating immune cells between the low- and high-risk groups. (**H**) Barplot showing the proportions of infiltrating immune cells in the low- and high-risk groups. (**I**) Heatmap for differences in the scores of immune cells between low- and high-risk groups.

To evaluate the immune landscape associated with the ireRNA-based signature, we calculated the stromal score, immune score and ESTIMATE score in both risk groups. As shown in Figures 5A–C, all immune-related scores were lower in the high-risk group compared to the low-risk group. The median scores were used to further stratify the patients into the respective high-score and low-score groups. As shown in Figures 5D–F, patients with high immune/ESTIMATE scores had significantly longer OS compared to those in the low-score group. In addition, we also compared the immune infiltration status in the high-risk and low-risk-groups using CIBERSORT. As shown in Figure 5G, the high-risk group had significantly lower proportions of infiltrating plasma cells, T cells CD8, T cells CD4 memory activated, T cells follicular helper and macrophages M1, and significantly higher proportions of NK cells resting, macrophages M0 and macrophages M2 compared to the low-risk group. The association between each immune cell population and the risk score is shown in Figures 5H,I.

### Functional analysis of the ireRNA-related signatures

**Figure 6:**
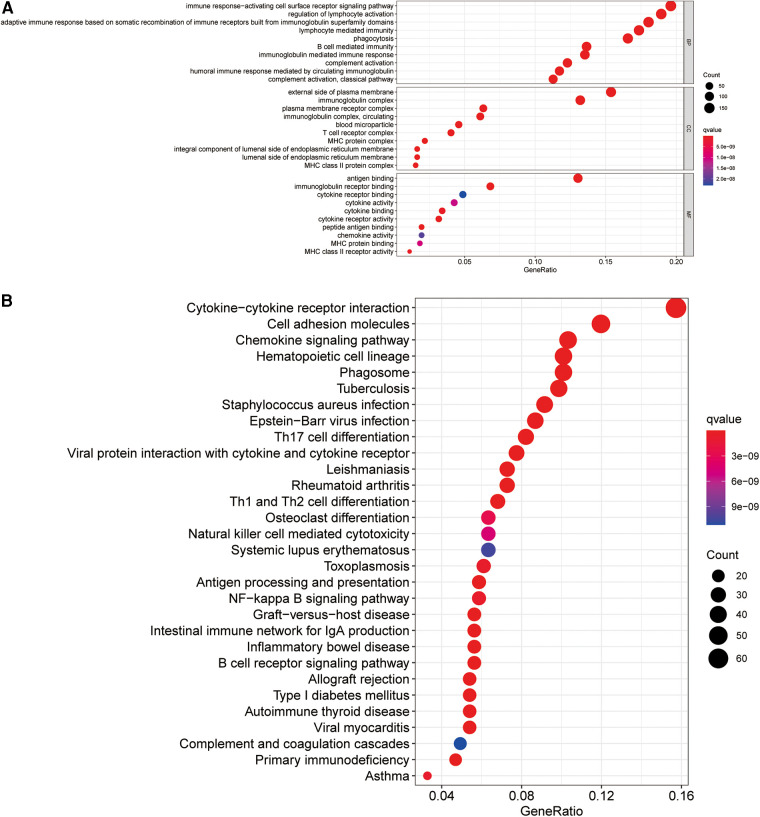
Functional analysis for the signature. (**A**) Significantly enriched biological processes in GO analysis. (**B**) Significantly enriched pathways in KEGG analysis.

To elucidate the potential biological processes and signaling pathways involving the ireRNA signature in cutaneous melanoma, we functionally annotated the 1,013 DEGs between the high-risk and low-risk groups (Supplementary Table S2) through GO and KEGG analyses. The genes in the high-risk group were mainly enriched in immune-related processes and pathways, including cytokine−cytokine receptor interaction, immune response−activating cell surface receptor signaling pathway, regulation of lymphocyte activation, adaptive immune response, etc. (Figures 6A,B). These results suggested that the prognostically relevant ireRNAs identified for cutaneous melanoma may influence tumor progression by regulating the immune microenvironment.

### Identifying the potential anti-cancer drug using cMap database

A total of 1,309 anti-cancer drug candidates were identified for cutaneous melanoma, of which corticosterone was ranked first and therefore may have the highest therapeutic potential.

## Discussion

Melanoma is the most lethal form of skin cancer and has the potential for metastasis at the early stage. Until recently, metastatic melanoma could evade nearly all attempts at therapy. Accurate biomarkers are essential for improving the design of therapies to increase survival. However, the currently used AJCC staging system cannot predict the prognosis of melanoma patients accurately or consistently due to the variability between pathologists, and the inability to identify tumors with high risk of metastasis at the early stage (3, 22). Thus, these problems warrant the development of novel prognostic markers. There is ample evidence suggesting that eRNAs regulate the transcriptional activation of target genes in human diseases, including cancer, which is indicative of their potential as therapeutic targets (23). The identification of eRNAs is a breakthrough in the field, enabling us to in-depth characterize the landscape of transcriptional circuitry in cancers. Moreover, eRNAs are increasingly realized to be involved in the immune response. Interestingly, cutaneous melanoma is one of the most immunogenic tumors. In this study, therefore, we focused on identifying ireRNAs with prognostic significance to construct a signature for cutaneous melanoma. Compared with single eRNA-analysis, this method combined eRNAs and immune had higher information content and could reflect the complex interaction of eRNAs and tumor immune that mediated melanoma development and progression.

We identified 33 ireRNAs, of which some are associated with immune functions and cancer progression. For example, HCP5 is aberrantly expressed in several different cancers and correlates with poor prognosis in patients with lung adenocarcinoma (24). In our study as well, HCP5 was identified as a survival-relevant eRNA in cutaneous melanoma. Furthermore, recent findings suggested that HCP5 was involved in adaptive and innate immune responses (25). Besides, Xu et al. found that LINC-PINT suppressed the tumorigenicity of melanoma by recruiting EZH2 to the promoter of target genes (26). Importantly, LINC-PINT has been proved to be a positive regulator of host innate immune responses, especially IFN signaling (27). LINC01094 expression predicted poor prognosis in patients with gastric cancer and was correlated with the macrophage infiltration (28). Moreover, one study showed that MIR100HG participated in the immune escape of gastric cancer cells (29). Taken together, these results demonstrated a significant relationship between identified eRNAs and immune. In this regard, these findings also verified the accuracy of the results of this present study. However, the molecular mechanisms of ireRNAs in melanoma remain largely unstudied.

The prognostic signature based on the OS-ireRNAs included AC009495.2, LINC02446, LINC00189, RSRP1, CUTALP, CMAHP and MOSMO, and accurately predicted the prognosis of melanoma patients, especially for those who survived for more than 3 years. Additionally, the ireRNA-related signature proved robust when validated in another cohort. More importantly, multivariate analysis verified the risk score as an independent prognostic factor. Thus, our study provided an additional accurate predictive tool to clinical practice, in order to provide support in treatment decision-making.

The enhancer elements are frequently dysregulated during cancer initiation and progression. The eRNAs are the most reliable predictor of enhancer activity, and may alter the expression of several key genes during cancer progression (30). Therefore, we explored the biological function of the established risk score in melanoma, and found significant enrichment of immune-related processes and pathways in the GO and KEGG analyses. This was not surprising given the fact that eRNAs are ubiquitously produced in response to immunological and other stimuli (18, 31, 32). Furthermore, since the eRNAs are associated with coding genes involved in immune-regulatory pathways (33), we characterized the immune landscape in melanoma based on the risk score. In this study, the high-risk patients had lower proportions of Macrophages M1 and higher proportions of Macrophages M2. Tumor-associated macrophages (TAMs) are the major immune components of the tumor microenvironment, oscillating between an M1, anti-cancer phenotype and an M2, tumor-promoting phenotype (34). An increased proportion of infiltrating TAMs is found in the melanoma microenvironment, specifically in the M2 phenotype, which favors neoplastic growth and dissemination (35, 36). Studies have confirmed that the enrichment of M2 was a poor indicator for the outcome of patients with melanoma (35, 37). Our results are in agreement with previous findings. CD8+ T cells are the primary effectors of the anti-tumor adaptive immune response, which not only inhibit tumor growth but also mediate responses to cancer immunotherapies (38). Furthermore, increased infiltration of CD8+ T cells has been linked to prolonged survival of cutaneous melanoma patients (39). Consistent with this, the high-risk patients in our cohorts had lower infiltration of CD8+ cells. In this regard, these results suggested that the identified signature was closely associated with the tumor immune in melanoma.

Increasing evidence points to the potential of eRNAs as therapeutic targets for cancers. In addition, eRNAs have been proved to have an essential role in mediating cancer cell drug response (40). Zhang et al. found that *NET1e* overexpression increased IC50 of Obatoclax and BEZ235 in breast cancer cells, indicating a direct role of eRNAs in drug response (19, 40). Therefore, we also screened for the drug candidates of melanoma, and identified corticosterone as a novel therapeutic drug. A recent study showed that stress-induced increase in corticosterone levels suppressed tumor growth in a model of malignant melanoma (41). It was worth mentioning that the antitumor effect is mainly through reducing recruitment of TAMs (41). As mentioned above, the infiltration of TAMs is an adverse prognostic factor for melanoma. Thus, there might be a crosstalk between tumor immune and the antitumor effects of corticosterone in melanoma. It will be an important future direction to illustrate the molecular mechanism of corticosterone suppressing melanoma growth and the influences on tumor immunity.

Notwithstanding the salient points of the present study, there were some limitations that ought to be considered. Firstly, due to the lack of datasets containing all the information needed for this analysis, we just analyzed the data from the TCGA cohort. Although we verified the signature in two separate subsets, the data was relatively insufficient. Furthermore, datasets for the analysis were all retrospective, and these results were not validated prospectively.

## Conclusion

In this present study, we constructed a prognostic signature for melanoma by integrating eRNAs and immune-related genes, which provided reliable information to better understand the mechanism of ireRNAs in the progression of melanoma. Moreover, we identified corticosterone as a potential antitumor-drug for melanoma, which warrants further research.

## Data Availability

The datasets presented in this study can be found in online repositories. The names of the repository/repositories and accession number(s) can be found in the article/Supplementary Material.
