# Supplementary figures and images for: Comprehensive transcriptomic analysis of immune-related eRNAs associated with prognosis and immune microenvironment in melanoma
Source: Front Surg. 2022 Sep 9;9:917061. doi: 10.3389/fsurg.2022.917061 (PMC9632973; doi:10.3389/fsurg.2022.917061)

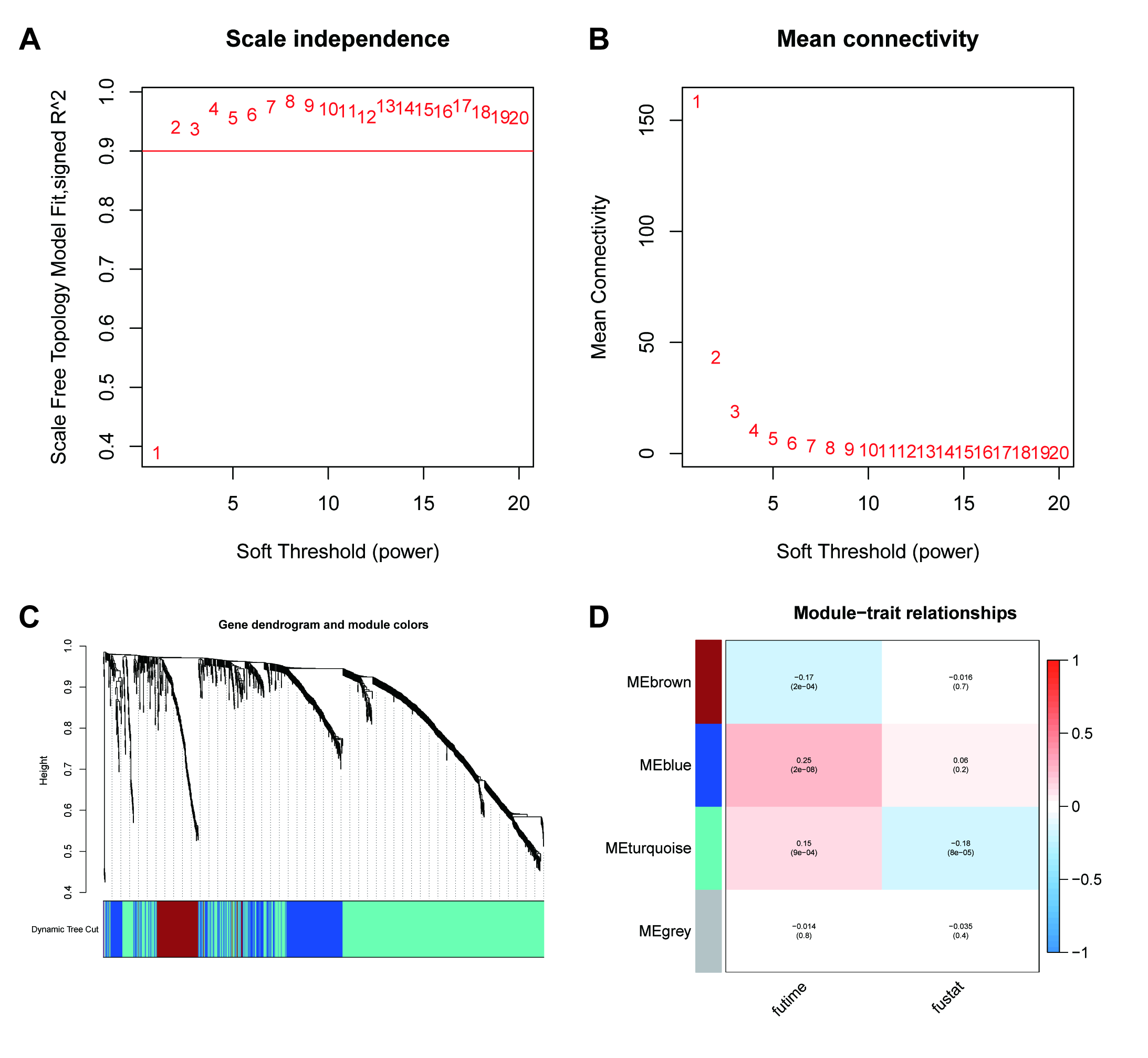

Supplement: Supplementary file 1 [file Image1.tif]
